# Risk Factors for Falls in Patients on Hemodialysis: A 12‐Month Prospective Study

**DOI:** 10.1111/hdi.70068

**Published:** 2026-03-10

**Authors:** Luciana Angélica da Silva de Jesus, Bruno Valle Pinheiro, Ana Clara Cattete Bainha, Rodrigo Schinniger Assun Garcia, Letícia Maria do Carmo Corrêa, Helena Faria Mucci, Erich Vidal Carvalho, Pelagia Koufaki, Leda Marília Fonseca Lucinda, Cristino Carneiro Oliveira, Maycon Moura Reboredo

**Affiliations:** ^1^ Research Group on Rehabilitation in Chronic Health Conditions—Federal University of Juiz de Fora Juiz de Fora Minas Gerais Brazil; ^2^ School of Medicine Federal University of Juiz de Fora Juiz de Fora Minas Gerais Brazil; ^3^ Centre of Health, Activity and Rehabilitation Research, School of Health Sciences Queen Margaret University Edinburgh Scotland UK; ^4^ Barbacena School of Medicine Barbacena Minas Gerais Brazil; ^5^ Department of Integrated Health Education, Physiotherapy Federal University of Espírito Santo Vitória Espírito Santo Brazil; ^6^ Postgraduate Research Program in Rehabilitation Sciences Federal University of Minas Gerais Belo Horizonte Minas Gerais Brazil

**Keywords:** accidental falls, end‐stage renal disease, hemodialysis, risk

## Abstract

**Background:**

Patients on hemodialysis have comorbidities and malnutrition and are subject to polypharmacy, which contributes to disability and sarcopenia. These conditions increase the risk of falls and are associated with fractures, morbidities, substantial costs, nursing home admissions, hospitalization, and mortality.

**Objective:**

This prospective study evaluated the association of physical function, postural balance, frailty, fear of falling, and quality of life with the occurrence and number of falls within a 12‐month interval in patients on hemodialysis.

**Methods:**

Patients were assessed for physical function (gait speed over 15 ft., timed up and go [TUG] test, 5‐repetition sit‐to‐stand [5‐STS] test, and handgrip strength), postural balance (Mini‐Balance Evaluation Systems Test [Mini‐BESTest]), frailty, fear of falling (Falls Efficacy Scale‐International [FES‐I]), and quality of life (36‐Item Short Form Health Survey [SF‐36]). Interviews were conducted for 12 months to monitor falls.

**Results:**

One hundred twelve patients were included and the incidence rate was 1.62 falls/person‐years. The occurrence of falls was associated with the TUG (OR: 1.24; 95% CI: 1.01–1.53) and 5‐STS (OR: 1.11, 95% CI: 1.02–1.21) performance and frailty (OR: 7.22, 95% CI: 1.71–30.50). The number of falls was associated with the gait speed (OR: 0.22; 95% CI: 0.06–0.77), TUG test results (OR: 1.37; 95% CI: 1.16–1.62), handgrip strength (OR: 0.95; 95% CI: 0.91–0.99), Mini‐BESTest (OR: 0.87; 95% CI: 0.78–0.96), frailty (OR: 4.43; 95% CI: 1.87–10.51), FES‐I score (OR: 1.11; 95% CI: 1.06–1.17), and SF‐36 scores in the physical functioning (OR: 0.98; 95% CI: 0.96–0.99), physical role (OR: 0.99; 95% CI: 0.98–0.99), and physical component summary (OR: 0.96; 95% CI: 0.92–0.99) domains.

**Conclusions:**

Patients undergoing hemodialysis have a higher incidence of falls. Falls are associated with physical function, postural balance, frailty, and quality of life.

## 1 Introduction

Patients with end‐stage kidney disease (ESKD) on hemodialysis have comorbidities and malnutrition and are subject to polypharmacy, which contributes to disability and sarcopenia [1]. Moreover, these patients present with symptoms, such as fatigue, pain, muscle cramps, difficulty concentrating, and dizziness, that lead to poor quality of life and impairment of personal, family, and social life [2]. These conditions increase the risk of falls and are associated with fractures, morbidities, substantial costs [1], nursing home admissions, hospitalization, and mortality [3, 4, 5, 6].

The occurrence of falls in patients with ESKD is also associated with hypotension, arrhythmias, and postural balance impairment caused by hemodialysis [4, 7]. A retrospective study in the United States of older patients with ESKD showed that the risk of serious fall injury was 1.6‐fold higher in the post‐dialysis period than during pre‐dialysis therapy initiation [8]. Bowling et al. found that a serious fall injury in the year before dialysis initiation is associated with a higher risk of additional serious fall injuries, hospitalization, skilled nursing facility admissions, and mortality in the first year of dialysis [9]. Additionally, patients with a serious fall injury in the year after hemodialysis initiation were more likely to have functional limitations. Other studies have shown that in the post‐dialysis period, patients have poor postural balance and a higher risk of falls [7].

Risk factors for falls can be classified as intrinsic (e.g., demographics, physical and psychological health, sensory impairment, and comorbidities) or extrinsic (e.g., personal care, environment, and living situations) [10]. Prospective studies including patients on hemodialysis have shown that demographic and clinical factors are associated with falls, such as age [6, 11, 12, 13], higher body weight [12], history of falls [14, 15, 16], hypotension [13, 15, 16], higher parathyroid hormone and C‐reactive protein levels [13, 15], higher number of comorbidities and oral prescribed drugs [6, 11, 16], and malnutrition [13]. Regarding physical function, a higher occurrence of falls is associated with reduced muscle mass [12], impaired postural balance, as assessed by static posturography [17], decreased step length [18], and poor performance in the 10 m walking test [6], Timed Up and Go (TUG) test [11], gait speed [18], Short Physical Performance Battery, and handgrip strength [13].

Moreover, the number of falls in patients undergoing hemodialysis is associated with frailty [19, 20], daily step counts [19], and poor physical performance [17, 19, 21, 22, 23]. A prospective study by Zanotto et al. demonstrated that the degree of cardiovascular dysregulation in the short‐term regulation of blood pressure predicts the number of falls in these patients, as evidenced by the predominance of self‐reported dizziness symptoms before falls [19].

However, few prospective studies have evaluated the association of the occurrence and number of falls with postural balance assessed using a physical function test, fear of falling, and quality of life in patients on hemodialysis. Furthermore, confirmation of previously reported factors linked to the risk of falls is necessary to reinforce their investigation in clinical practice. The primary objective of this study was to evaluate the association of physical function, postural balance, frailty, fear of falling, and quality of life with the occurrence and number of falls within a 12 months in patients on hemodialysis. The secondary objective was to evaluate the incidence of falls in these patients.

## 2 Materials and Methods

### 2.1 Ethics Approval

This study followed ethical principles of the Declaration of Helsinki and was approved by the University Hospital of the Federal University of Juiz de Fora Research Ethics Committee (number: 4.106.335/2020) and Barbacena School of Medicine Research Ethics Committee (number: 3.741.115/2019). All patients gave written informed consent.

### 2.2 Study Design

This 12‐month prospective study was conducted between July 2021 and May 2023. Patients were recruited from the Nephrology Unit of the University Hospital of the Federal University of Juiz de Fora (Juiz de Fora, MG, Brazil) and the Pro‐Renal Center (Barbacena, MG, Brazil).

Initial patient evaluations included physical function (gait speed over 15 ft., TUG test, five‐repetition sit‐to‐stand [5‐STS] test, and handgrip strength) and postural balance (Mini‐Balance Evaluation System Test [Mini‐BESTest]). These tests were performed on two different days, before the start of the second or third dialysis session of the week. During the dialysis session, the patients were interviewed to assess frailty (modified Fried phenotype), fear of falling (Falls Efficacy Scale‐International [FES‐I]), and quality of life (36‐Item Short Form Health Survey [SF‐36]). Following this initial assessment, the occurrence of falls was monitored using a monthly standardized evaluation for 12 months. A previously trained team performed all assessments.

This study was conducted in accordance with the STROBE (Strengthening the Reporting of Observational Studies in Epidemiology) guidelines.

### 2.3 Patients

The sample was recruited by convenience sampling and included patients with ESKD, aged ≥ 18 years, and who were undergoing hemodialysis treatment three times per week, totaling 12 h weekly, for at least 3 months. The exclusion criteria were as follows: musculoskeletal or osteoarticular disorders, wheelchair use, assisted gait, uncorrected visual impairments, psychiatric or cognitive disorders, presence of severe and unstable comorbidities (e.g., angina, heart failure, chronic respiratory diseases, acute systemic infection, or myocardial infarction), and hospitalization in the past 3 months.

### 2.4 Measurements

#### 2.4.1 Sociodemographic, Clinical, and Laboratory Data

Age, sex, educational level, time on dialysis, body mass index, comorbidities, history of falls (assessed in the last 12 months), and laboratory data (hemodialysis efficiency index and hemoglobin, albumin, calcium, phosphorus, and parathyroid hormone levels) were collected from medical records or patient interviews.

#### 2.4.2 Physical Function

Gait speed was measured using a 15 ft. walk (4.57 m). Patients walked at their usual speed on an 8.57 m course using a 2 m acceleration zone, 4.57 m timing area, and 2 m deceleration zone. The best test (m/s) among the three trials was used for analysis, with a higher value indicating better performance.

The TUG test recorded the time required for the patient to stand, walk to a line for a distance of 3 m, turn around, walk back to the chair, and sit. The patients were advised to walk as quickly as possible, safely, and comfortably. The shortest time (s) was recorded between two trials, and a longer time suggested poor mobility.

In the 5‐STS test, the patients were instructed to stand from a seated position using a standard‐height chair and then sit down with their arms folded across their chest. The time (s) to perform five full repetitions was recorded, and a higher time reflected worse strength.

Handgrip strength was assessed using a hydraulic hand dynamometer (Jamar Patterson Medical Ltd., USA) on the non‐fistula arm. Three attempts were conducted, and the best strength (kgf) was used. A higher value indicated better performance.

#### 2.4.3 Postural Balance

The Mini‐BESTest consisted of 14 items organized into four categories (anticipatory postural control, reactive postural control, sensory orientation, and dynamic gait). The score ranged from 0 to 28 points, with higher scores indicating better postural balance performance.

#### 2.4.4 Frailty

Modified Fried phenotype has five components: unintentional weight loss of ≥ 4.5 kg in the previous year; weakness, with a handgrip strength of < 27 kgf for males and < 16 kgf for females; exhaustion, assessed using two questions from the Center for Epidemiological Studies Depression scale; low gait speed over 15 ft. (≤ 0.8 m/s); and low physical activity level evaluated using a cutoff value of 53 for the adjusted activity score of the Human Activity Profile questionnaire. Patients were classified as frail if they met at least three of these criteria.

#### 2.4.5 Fear of Falling

The FES‐I is a 16‐item questionnaire that evaluates concerns about falling during various daily life and social activities. The level of concern for each question was scored from 1 to 4, which indicated not at all, somewhat, fairly, and very concerned. A higher score indicated a higher fear of falling.

#### 2.4.6 Quality of Life

The SF‐36 questionnaire consists of 36 items that evaluate physical functioning, physical role, pain, general health, vitality, social functioning, emotional role, and mental health. These eight dimensions were aggregated into physical and mental component summaries. Higher scores reflect a better quality of life.

#### 2.4.7 Monitoring Falls

A fall was defined as “an unexpected event in which the individual came to rest on the ground, floor, or lower level” [24]. Monthly interviews were conducted for 12 months during the dialysis sessions to record falls and investigate their characteristics. Patients who did not experience any falls and those who fell at least once were classified as non‐fallers and fallers, respectively. The number of falls was recorded.

### 2.5 Statistical Analysis

The sample size estimation was based on the number of variables intended to be included in the multivariate regression models (nine variables) as independent variables, with at least 10 patients for each factor.

The data normality was analyzed using the Shapiro–Wilk test. Numerical variables are expressed as the mean and standard deviation or median [25th–75th quartiles] for normal and non‐normal distributions, respectively. Categorical variables are expressed as the number of patients (percentage). Missing data are presented in Table S1; imputation was not performed.

The incidence of falls was calculated as the ratio of the number of patients who fell to the number of patients who were followed up. In the incidence rate, the numerator represented the number of the first recording of falls, and the denominator was the sum of the time periods of observation for all patients at risk during the study follow‐up, recorded in person‐years. Comparisons between fallers and non‐fallers were performed using the Student's *t*‐test, Mann–Whitney test, or chi‐square test.

A univariate logistic regression model was used to evaluate the association between fall occurrence (fallers and non‐fallers) and physical function, postural balance, frailty, fear of falling, and quality of life. Each significantly associated variable was considered an independent variable in a multivariate logistic regression model. The model was constructed with the occurrence of falls as the dependent variable adjusted for potential confounders, including differences in sociodemographic, clinical, and laboratory data found in the comparisons between fallers and non‐fallers.

A univariate negative binomial regression model was used to evaluate the associations between the number of falls and physical function, postural balance, frailty, fear of falling, and quality of life. Each significantly associated variable was considered an independent variable in a multivariate negative binomial regression model constructed with the number of falls as the dependent variable adjusted for potential confounders. These confounders were defined in a univariate negative binomial regression, with the number of falls as the dependent variable and sociodemographic, clinical, and laboratory data as the independent variables.

Statistical analyses were performed using SPSS software version 22.0. Statistical significance was set at *p* < 0.05.

## 3 Results

**FIGURE 1:**
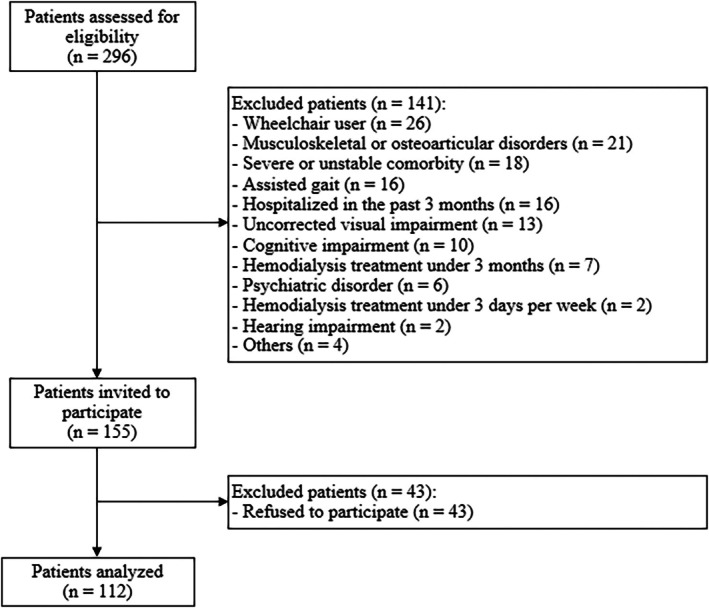
Flowchart of patients' selection.

Of the 296 patients assessed for eligibility, 184 were excluded, and 112 were followed up for 12 months (Figure 1). The incidence of falls was 31.3%. Table 1 shows the sociodemographic, clinical, and laboratory characteristics of patients. Patients classified as fallers had a higher body mass index and calcium levels than those classified as non‐fallers. Additionally, fallers and non‐fallers had no significant differences in medication prescriptions (Table S2).

**TABLE 1:** Sociodemographic, clinical, and laboratory characteristics, and physical function, postural balance, frailty, fear of falling and quality of life of the patients.

Variables	Total (*n* = 112)	Non‐fallers (*n* = 77)	Fallers (*n* = 35)	*p*
Age (y)	58.7 ± 12.9	57.9 ± 13.8	60.5 ± 11.0	0.335
Male, *n* (%)	63 (56.3)	44 (57.1)	19 (54.3)	0.778
Educational level (*y*)	7.0 (5.0–11.0)	6.0 (5.0–10.0)	7.5 (5.0–11.0)	0.780
Family income (US$)	524.2 (286.5–653.5)	524.2 (302.7–648.5)	524.2 (262.1–759.9)	0.894
Time on HD (m)	30.5 (13.0–54.5)	29.0 (12.5–49.5)	35.0 (13.0–62.0)	0.653
Body mass index (kg/m^2^)	24.4 (21.9–28.0)	23.9 (21.4–26.8)	25.9 (23.4–30.5)	0.026
Obesity, *n* %	18 (16.1)	8 (10.4)	10 (28.6)	0.015
History of fall, *n* (%)	53 (47.3)	37 (48.1)	16 (45.7)	0.818
History of fracture, *n* (%)	17 (15.2)	11 (14.3)	6 (17.1)	0.696
Comorbidities, *n* (%)				
Hypertension	108 (96.4)	75 (97.4)	33 (94.3)	0.410
Cardiovascular disease	72 (64.3)	53 (68.8)	19 (54.3)	0.136
Diabetes mellitus	48 (42.9)	31 (40.3)	17 (48.6)	0.410
Neurologic disease	19 (17.0)	13 (16.9)	6 (17.1)	0.973
Hemodialysis efficiency index	1.5 (1.3–1.7)	1.5 [1.3–1.6]	1.5 (1.4–1.7)	0.142
Hemoglobin (g/dL)	10.6 ± 2.2	10.3 ± 2.3	11.2 ± 1.9	0.056
Hemoglobin < 10 g/dL, *n* (%)	45 (40.2)	11 (31.4)	34 (44.2)	0.203
Albumin (g/dL)	3.9 (3.7–4.1)	3.9 (3.6–4.1)	3.9 (3.7–4.2)	0.444
Calcium (mg/dL)	9.1 ± 0.9	8.9 ± 0.8	9.5 ± 0.7	0.001
Normocalcemia, *n* (%)	62 (55.4)	36 (46.8)	26 (74.3)	0.001
Hypocalcemia, *n* (%)	41 (36.6)	37 (48.1)	4 (11.4)	0.001
Hypercalcemia, *n* (%)	9 (8.0)	4 (5.2)	5 (14.3)	0.001
Phosphorous (mg/dl)	5.2 (4.3–6.3)	5.2 (4.3–5.9)	5.3 (4.3–6.7)	0.303
Parathyroid hormone (pg/mL)	475.7 (244.8–848.8)	534.3 (230.4–861.7)	409.6 (292.1–671.9)	0.457
Gait speed (m/s)	1.3 ± 0.9	1.3 ± 0.3	1.3 ± 0.4	0.866
TUG (s)	8.2 (6.7–9.4)	8.1 (6.4–9.0)	8.7 (7.6–10.7)	0.017
Handgrip strength (kgf)	26.0 (20.0–35.0)	28.0 (22.0–36.0)	22.0 (16.0–34.0)	0.038
5‐STS (s)	13.4 (10.9–16.9)	12.4 (10.5–16.4)	14.9 (12.2–20.4)	0.040
Mini‐BESTest score	22.0 (20.0–24.0)	22.0 (20.3–24.8)	21.0 (20.0–24.0)	0.192
Frailty, *n* (%)	14 (13.0)	4 (5.5)	10 (28.6)	0.001
FES‐I score	23.0 (18.0–29.8)	21.0 (18.0–29.0)	26.0 (18.0–32.0)	0.116
SF‐36 questionnaire score				
Physical functioning	70.0 (50.0–93.8)	75.0 (52.5–95.0)	65.0 (45.0–90.0)	0.119
Physical role	50.0 (0–93.8)	50.0 (0–75.0)	25.0 (0–100)	0.702
Pain	62.0 (41.0–100)	62.0 (41.0–92.0)	61.0 (41.0–100)	0.934
General health	52.0 (37.0–72.0)	52.0 (36.0–72.0)	57.0 (42.0–72.0)	0.511
Vitality	61.2 ± 22.8	62.8 ± 21.1	57.6 ± 26.2	0.264
Social functioning	75.0 (50.0–100)	75.0 (43.8–100)	75.0 (62.5–100)	0.189
Emotional role	33.3 (0–100)	33.3 (0–100)	33.3 (0–100)	0.901
Mental health	68.0 (53.0–87.0)	72.0 (56.0–88.0)	64.0 (44.0–84.0)	0.219
Physical component summary	42.9 ± 9.3	43.2 ± 9.1	42.5 ± 9.9	0.729
Mental component summary	23.7 (15.7–37.8)	37.6 (25.1–50.2)	39.7 (20.8–54.8)	0.826

*Note:* Values are expressed as the mean ± standard deviation, median (25%–75% quartile) or number of patient (percentage).

Abbreviations: 5‐STS = 5‐repetition sit‐to‐stand test; FES‐I = falls efficacy scale‐international; Mini‐BESTest = mini balance evaluation systems test; SF‐36 = 36‐item short form health survey; TUG = Timed up and go.

The incidence rate was 1.62 (95% CI: 1.03–2.22) falls/person‐years. Falls occurred mainly on non‐dialysis days and at patients' homes. The most prevalent symptoms reported before a fall were fatigue and weakness. Approximately 20% of falls caused an injury, and the most prevalent consequences of falls were more caution and greater concern about falling (Table S3).

The physical function, postural balance, frailty, fear of falling, and quality of life of the patients are shown in Table 1. Fallers had poor performance on the TUG, 5‐STS, and handgrip strength tests and a higher prevalence of frailty than non‐fallers.

Univariate logistic regression analysis showed that the performance on the TUG and 5‐STS tests and the presence of frailty were significantly associated with the occurrence of falls. After adjusting for potential confounders in the multivariate logistic regression analysis, these associations remained significant (Table 2). Notably, the inclusion of hemoglobin < 10 g/dL in the multivariate logistic regression model did not materially change the associations (Table S5).

**TABLE 2:** Univariate and multivariate logistic regression models including the occurrence of falls as dependent variable and physical function and frailty as independent variables (*n* = 112).

Variables	Unadjusted analysis		Adjusted analysis^a^	
	OR (95% CI)	*p*	OR (95% CI)	*p*
TUG (s)	1.23 (1.02–1.49)	0.030	1.24 (1.01–1.53)	0.046
Handgrip strength (kgf)	0.96 (0.93–1.01)	0.089		
5‐STS (s)	1.08 (1.00–1.17)	0.045	1.11 (1.02–1.21)	0.016
Frailty	6.90 (1.98–24.0)	0.002	7.22 (1.71–30.50)	0.007

Abbreviations: 5‐STS = 5‐repetition sit‐to‐stand test; 95% CI = 95% confidence interval; OR = odds ratio; TUG = Timed up and go.

^a^ Body mass index categorized as obesity (greater than or equal to 30 kg/m^2^) and calcium levels categorized as normocalcemia (8.8–10.4 mg/dL), hypocalcemia (< 8.8 mg/dL), and hypercalcemia (> 10.4 mg/dL).

In the multivariate negative binomial regression, after adjusting for potential confounders, the gait speed, TUG results, handgrip strength, Mini‐BESTest scores, presence of frailty, FES‐I scores, and scores on the SF‐36 questionnaire (physical functioning, physical role, and physical component summary) were significantly associated with the number of falls (Table S4 and Table 3). These associations remained significant after further adjustment for hemoglobin < 10 g/dL (Table S6).

**TABLE 3:** Univariate and multivariate negative binomial regression models including the number of falls as dependent variable and physical function, postural balance, frailty, fear of falling, and quality of life as independent variables (*n* = 112).

Variables	Unadjusted analysis		Adjusted analysis^a^	
	OR (95% CI)	*p*	OR (95% CI)	*p*
Gait speed (m/s)	0.35 (0.13–0.89)	0.028	0.22 (0.06–0.77)	0.018
TUG (s)	1.15 (1.03–1.28)	0.016	1.37 (1.16–1.62)	< 0.001
Handgrip strength (kgf)	0.96 (0.93–0.98)	0.001	0.95 (0.91–0.99)	0.016
5‐STS (s)	1.01 (0.97–1.06)	0.553		
Mini‐BESTest score	0.83 (0.76–0.90)	< 0.001	0.87 (0.78–0.96)	0.004
Frailty	6.02 (3.07–11.82)	< 0.001	4.43 (1.87–10.51)	0.001
FES‐I score	1.12 (1.08–1.16)	< 0.001	1.11 (1.06–1.17)	< 0.001
SF‐36 questionnaire score				
Physical functioning	0.97 (0.96–0.98)	< 0.001	0.98 (0.96–0.99)	0.008
Physical role	0.98 (0.98–0.99)	< 0.001	0.99 (0.98–0.99)	0.021
Pain	0.99 (0.98–0.99)	0.035	1.01 (0.99–1.02)	0.724
General health	0.98 (0.96–0.99)	< 0.001	0.99 (0.98–1.01)	0.561
Vitality	0.98 (0.97–0.99)	< 0.001	0.99 (0.97–1.01)	0.094
Social functioning	1.01 (0.99–1.02)	0.140		
Emotional role	0.99 (0.99–1.01)	0.397		
Mental health	0.98 (0.97–0.99)	0.001	0.98 (0.97–1.01)	0.052
Physical component summary	0.93 (0.91–0.96)	< 0.001	0.96 (0.92–0.99)	0.048
Mental component summary	0.99 (0.98–1.01)	0.325		

Abbreviations: 5‐STS = 5‐repetition sit‐to‐stand test; 95% CI = 95% confidence interval; FES‐I = Falls Efficacy Scale‐International; Mini‐BESTest = Mini Balance Evaluation Systems Test; OR = odds ratio; SF‐36 = 36‐Item Short Form Health Survey; TUG = Timed up and go.

^a^ Age, gender, educational level, body mass index, body mass index categorized as obesity (greater than or equal to 30 kg/m^2^), history of fall, cardiovascular disease, and calcium levels categorized as normocalcemia (8.8–10.4 mg/dL), hypocalcemia (< 8.8 mg/dL), and hypercalcemia (> 10.4 mg/dL).

## 4 Discussion and Conclusions

This study evaluated the factors associated with the occurrence and number of falls in patients undergoing hemodialysis. The main findings of this study were as follows: (I) the incidence of falls was 31.3%, and the incidence rate was 1.62 falls/person‐years; (II) poor performance in the TUG and 5‐STS tests and the presence of frailty were significantly associated with the occurrence of falls; (III) a higher number of falls was associated with poor performance in the TUG test, the presence of frailty, and worse FES‐I scores; a lower number of falls was associated with better gait speed, handgrip strength, and Mini‐BESTest and SF‐36 questionnaire scores.

The incidence and incidence rate of falls in the present study were similar to those described in other studies of patients on hemodialysis [16, 17, 20]. However, the incidence of falls ranges from 26.3% to 55% [13, 14, 15, 16, 20], and the incidence rate ranges from 0.31 to 1.60 falls/person‐years [6, 11, 14, 16, 21, 22] in these patients. Variations in the incidence and incidence rate of falls among studies could be associated with differences in patient age and follow‐up periods. In this context, a prospective study of patients on hemodialysis showed incidence rates of 1.76 and 0.13 falls/patient‐years in older patients (> 65 years) and younger patients, respectively [15]. The high incidence of falls in patients on hemodialysis could be attributed to polypharmacy, complications of diabetes mellitus, orthostatic hypotension (especially when the dry‐weight is underestimated), vitamin D deficiency, sarcopenia, and anemia [1]. Moreover, the impairment of physical function increases the risk of falls in these patients [13, 19, 22].

In the present study, patients classified as fallers performed poorly in the TUG, 5‐STS, and handgrip strength tests compared to non‐fallers, but only the times required to perform the 5‐STS and TUG tests were associated with the occurrence of falls. Similarly, a prospective study of patients on hemodialysis showed that fallers had lower muscle handgrip strengths and physical functions assessed using the Short Physical Performance Battery than non‐fallers [21]. In contrast, Liang et al. demonstrated that moderately and severely abnormal TUG performance was an independent risk factor for falls in patients on hemodialysis [11]. Zanotto et al. reported that the handgrip strength and TUG test had diagnostic accuracy in discriminating patients on hemodialysis with a history of falls [25].

An important finding of this study was that frailty increased the risk of falls by 7.22 times. In another longitudinal study conducted on patients on hemodialysis, Delgado et al. showed that frailty increased the risk of a first fall or fracture by 60% [26]. Frailty is a complex syndrome that affects the physical, sensory, psychological, and social domains [2]. Furthermore, frailty is associated with adverse outcomes such as a decline in physical function, social isolation, morbidity, and mortality [2].

Another finding of this study is the association between a higher number of falls, poor performance on the TUG test, and the presence of frailty. Moreover, improved gait speed and handgrip strength are associated with fewer falls. Similarly, Shirai et al. found that poor performance on the TUG test before and after hemodialysis sessions was independently associated with a higher number of falls [22]. Another study demonstrated that frailty can independently predict a 3.1‐fold higher number of falls [20]. Zanotto et al. found that better gait speed and handgrip strength are associated with a lower number of falls and that the presence of cardiovascular dysregulation, as evaluated by baroreflex function and hemodynamic variables, is associated with a higher number of falls [19]. Additionally, lower handgrip strength and physical ability, according to the Short Physical Performance Battery score, are independently associated with a higher fall frequency [21].

To the best of our knowledge, this is the first study to investigate the association between the number of falls and postural balance using the Mini‐BESTest in patients on hemodialysis. The Mini‐BESTest assesses different aspects of dynamic postural balance (i.e., anticipatory and reactive postural control, sensory orientation, and dynamic gait) and can be performed easily [27]. We found that a 1‐point increase in the Mini‐BESTest score reduces the number of falls by 16%. Similar results were found in a study of older adults, in which a 1‐point reduction in the Mini‐BESTest score increased the risk of falls from 14% to 34% [28].

Interestingly, this study was also the first to show that a higher fear of falling is associated with a higher number of falls and that a better quality of life is associated with a lower number of falls. Some studies have demonstrated an association between a history of falls, fear of falling, and quality of life in these patients [14, 25, 29]. In a previous study, we showed that a higher fear of falling evaluated by the FES‐I is associated with a reduced quality of life in patients on hemodialysis [29]. In a prospective study of patients on dialysis, 68% of fallers reported limiting activities because of a fear of falling compared to 42% of non‐fallers, reflecting the impact of falls on the quality of life [21]. Moreover, Zanotto et al. found that the Falls Efficacy Scale score, physical functioning, and physical component summary of the SF‐36 can predict the risk of falls in patients undergoing hemodialysis [25].

From a clinical perspective, considering that the occurrence and number of falls are associated with modifiable risk factors in patients on hemodialysis (e.g., physical function, postural balance, fear of falling, and quality of life), our results can contribute to guiding interventions to prevent falls. Moreover, fall prevention in patients with ESKD includes a review of the medications, better glycemic control, vitamin D deficiency treatment, exercise training, staff and patient education, and environmental reconfiguration [1, 30].

This study had several strengths and limitations. The prospective design of this study allowed the assessment of different factors associated with the occurrence and number of falls among patients undergoing hemodialysis. Additionally, discriminating between the factors associated with the occurrence or number of falls is crucial for understanding the different aspects of fall scenarios in patients with ESKD, as single versus multiple falls require distinct prevention strategies. Nevertheless, this study had several limitations, including convenience sampling. Although this study was conducted in two dialysis units with broad inclusion criteria, our results cannot be generalized to all patients undergoing hemodialysis. Moreover, potential recall bias must be considered in fall assessments; however, monthly interviews with patients in this study may have mitigated this bias. Considering that the occurrence of falls is multifactorial, other prospective studies should be conducted to verify extrinsic factors such as personal care, environment, and social conditions [10]. Finally, neurological function assessments were not performed in this study. Considering that long‐term dialysis is associated with neurological complications involving both the central and peripheral nervous systems, the absence of neurological assessment may have affected the interpretation of our findings.

In conclusion, patients undergoing hemodialysis have a higher incidence of falls. Falls are associated with poor physical function and frailty. Additionally, physical function, postural balance, frailty, fear of falling, and quality of life are associated with the number of falls. Our results contribute to the understanding of the factors associated with falls and can be used to implement interventions to prevent falls in patients undergoing hemodialysis.

## Funding

This study was supported by a Research Grant from Fundação de Amparo à Pesquisa do Estado de Minas Gerais—FAPEMIG [grant numbers APQ 02229, 2018] and Coordenação de Aperfeiçoamento de Pessoal de Nível Superior—CAPES [grant numbers 001, 2023]. M.M.R. was supported by the National Council for Scientific and Technological Development—CNPq.

## Conflicts of Interest

The authors declare no conflicts of interest.

## Supporting information


**Table S1:** Missing data.
**Table S2:** Medication prescription of the patients.
**Table S3:** Characterization of falls.
**Table S4:** Univariate negative binomial regression including the number of falls as dependent variable, and sociodemographic, clinical and laboratory characteristics as independent variables (*n* = 112).
**Table S5:** Univariate and multivariate logistic regression models including the occurrence of falls as dependent variable and physical function and frailty as independent variables (*n* = 112).
**Table S6:** Univariate and multivariate negative binomial regression models including the number of falls as dependent variable and physical function, postural balance, frailty, fear of falling, and quality of life as independent variables (*n* = 112).

## Data Availability

The data that support the findings of this study are available from the corresponding author upon reasonable request.
