# Supplementary material for: Risk Factors for Falls in Patients on Hemodialysis: A 12‐Month Prospective Study
Source: Hemodial Int. 2026 Mar 10;30(3):591–9. doi: 10.1111/hdi.70068 (PMC13350521; doi:10.1111/hdi.70068)
Supplement: Supplementary file 1 — Table S1: Missing data. Table S2: Medication prescription of the patients. Table S3: Characterization of falls. Table S4: Univariate negative binomial regression including the number of falls as dependent variable, and sociodemographic, clinical and laboratory characteristics as independent variables (n = 112). Table S5: Univariate and multivariate logistic regression models including the occurrence of falls as dependent variable and physical function and frailty as independent variables (n = 112). Table S6: Univariate and multivariate negative binomial regression models including the number of falls as dependent variable and physical function, postural balance, frailty, fear of falling, and quality of life as independent variables (n = 112). [file HDI-30-591-s001.docx]

**Risk factors for falls in patients on hemodialysis: a 12-month prospective study**

**Supplementary Table 1.** Missing data.

| **Variables** | **Total (n = 112)** | **Non-fallers (n = 77)** | **Fallers (n = 35)** |
| --- | --- | --- | --- |
| Educational level | 2 (1.8) | 1 (1.3) | 1 (2.9) |
| Family income | 4 (3.6) | 2 (2.6) | 2 (5.7) |
| Medication classes | 6 (5.4) | 5 (6.5) | 1 (2.9) |
| Hemodialysis efficiency index | 3 (2.7) | 3 (3.9) | 0 (0) |
| TUG (s) | 7 (6.3) | 6 (7.8) | 1 (2.9) |
| Handgrip strength (kgf) | 3 (2.7) | 3 (3.9) | 0 (0) |
| 5-STS (s) | 12 (10.7) | 8 (10.4) | 4 (11.4) |
| Mini-BESTest score | 3 (2.7) | 1 (1.3) | 2 (5.7) |
| Frailty | 4 (3.6) | 4 (5.2) | 0 (0) |

Values are expressed as number of patient (percentage)

TUG = Timed up and go, 5-STS = 5-repetition sit-to-stand test, Mini-BESTest = Mini Balance Evaluation Systems Test

**Supplementary Table 2.** Medication prescription of the patients.

| **Variables** | **Total (n = 112)** | **Non-fallers (n = 77)** | **Fallers (n = 35)** | ***p* value** |
| --- | --- | --- | --- | --- |
| Beta blockers, n (%) | 64 (60.4) | 47 (65.3) | 17 (50.0) | 0.133 |
| Calcium channel blocker, n (%) | 50 (47.2) | 34 (47.2) | 16 (47.1) | 0.987 |
| Angiotensin II-receptor antagonists, n (%) | 37 (34.9) | 26 (36.1) | 11 (32.4) | 0.705 |
| Diuretics, n (%) | 40 (37.7) | 28 (38.9) | 12 (35.3) | 0.722 |
| Vasodilator, n (%) | 25 (23.6) | 20 (27.8) | 5 (14.7) | 0.139 |
| Anti-inflammatories, n (%) | 25 (23.6) | 15 (20.8) | 10 (29.4) | 0.332 |
| Central action blockers, n (%) | 14 (13.2) | 10 (13.9) | 4 (11.8) | 0.763 |
| Angiotensin-converting enzyme inhibitors, n (%) | 8 (7.5) | 7 (9.7) | 1 (2.9) | 0.217 |
| Benzodiazepine, n (%) | 8 (7.5) | 4 (5.6) | 4 (11.8) | 0.259 |

Values are expressed as number of patient (percentage)

**Supplementary Table 3.** Characterization of falls.

| **Characteristic** | **Events (%)** |
| --- | --- |
| Moment of the fall |  |
| Nondialysis day | 53 (48.6) |
| Dialysis day before hemodialysis | 33 (30.0) |
| Dialysis day after hemodialysis | 23 (20.9) |
| Location of the fall |  |
| Home | 71 (65.1) |
| Street | 29 (26.6) |
| Dialysis unit | 9 (8.3) |
| Perceived Symptoms before the fall |  |
| Fatigue | 34 (31.2) |
| Weakness | 31 (28.4) |
| Dizziness | 21 (19.3) |
| Fainting | 2 (1.8) |
| Complications of the fall |  |
| Injury | 25 (22.7) |
| Needed care | 16 (14.5) |
| Death | 1 (0.9) |
| Consequences of the fall |  |
| More caution | 77 (70.6) |
| Greater concern about falling | 63 (57.8) |
| Modification of the location to prevent another fall | 24 (22.0) |
| Stopped doing any activity | 14 (12.8) |
| Use of a walking aid | 1 (0.9) |

**Supplementary Table 4.** Univariate negative binomial regression including the number of falls as dependent variable, and sociodemographic, clinical and laboratory characteristics as independent variables (n = 112).

| **Variables** | **OR (95% CI)** | ***p* value** |
| --- | --- | --- |
| Age (y) | 0.98 (0.95 - 0.99) | 0.037 |
| Female | 2.25 (1.31 - 3.86) | 0.003 |
| Educational level (y) | 1.18 (1.09 - 1.27) | <0.001 |
| Family income (US$) | 1.00 (0.99 - 1.00) | 0.725 |
| Time on HD (m) | 1.02 (0.99 - 1.01) | 0.344 |
| Body mass index (kg/m²) | 1.10 (1.05 - 1.15) | <0.001 |
| Obesity | 2.98 (1.58 - 5.64) | 0.001 |
| History of fall | 1.95 (1.14 - 3.33) | 0.015 |
| History of fracture | 0.75 (0.35 - 1.63) | 0.465 |
| Comorbidities |  |  |
| Hypertension | 2.00 (0.36 - 11.15) | 0.429 |
| Cardiovascular disease | 0.32 (0.18 - 0.55) | <0.001 |
| Diabetes mellitus | 1.33 (0.79 - 2.26) | 0.286 |
| Neurologic disease | 1.22 (0.62 - 2.41) | 0.560 |
| Hemodialysis efficiency index | 2.17 (0.89 - 5.28) | 0.088 |
| Hemoglobin (g/dL) | 1.00 (0.95 - 1.27) | 0.190 |
| Hemoglobin < 10g/dL | 0.558 (0.32 - 0.98) | 0.043 |
| Albumin (g/dL) | 1.38 (0.64 - 3.01) | 0.413 |
| Calcium (mg/dL) | 2.72 (1.90 - 3.89) | <0.001 |
| Classification of calcemia |  |  |
| Normal calcemia (8.8 - 10.4 mg/dL) | Reference |  |
| Hypocalcemia (< 8.8 mg/dL) | 0.17 (0.07 - 0.40) | <0.001 |
| Hypercalcemia (>10.4 mg/dL) | 4.37 (1.96 - 9.77) | <0.001 |
| Phosphorous (mg/dl) | 1.18 (0.99 - 1.40) | 0.052 |
| Parathyroid hormone (pg/mL) | 0.99 (0.99 - 1.00) | 0.083 |

OR = odds ratio, 95% CI = 95% confidence interval

**Supplementary Table 5.** Univariate and multivariate logistic regression models including the occurrence of falls as dependent variable and physical function and frailty as independent variables (n = 112).

|  | **Unadjusted analysis** | | **Adjusted analysis*** |  |
| --- | --- | --- | --- | --- |
| **Variables** | **OR (95% CI)** | ***p* value** | **OR (95% CI)** | ***p* value** |
| TUG (s) | 1.23 (1.02 - 1.49) | 0.030 | 1.29 (1.02 - 1.62) | 0.033 |
| Handgrip strength (kgf) | 0.96 (0.93 - 1.01) | 0.089 |  |  |
| 5-STS (s) | 1.08 (1.00 - 1.17) | 0.045 | 1.13 (1.03 - 1.23) | 0.009 |
| Frailty | 6.90 (1.98 - 24.0) | 0.002 | 4.25 (1.20 - 15.05) | 0.025 |
| OR = odds ratio, 95% CI = 95% confidence interval  *body mass index categorized as obesity (greater than or equal to 30 kg/m²), hemoglobin < 10 g/dL, and calcium levels categorized as normocalcemia (8.8 - 10.4 mg/dL), hypocalcemia (< 8.8 mg/dL), and hypercalcemia (>10.4 mg/dL)  TUG = Timed up and go, 5-STS = 5-repetition sit-to-stand test | | | | |

**Supplementary Table 6.** Univariate and multivariate negative binomial regression models including the number of falls as dependent variable and physical function, postural balance, frailty, fear of falling, and quality of life as independent variables (n = 112).

|  | **Unadjusted analysis** | | **Adjusted analysis*** | |
| --- | --- | --- | --- | --- |
| **Variables** | **OR (95% CI)** | ***p* value** | **OR (95% CI)** | ***p* value** |
| Gait speed (m/s) | 0.35 (0.13 - 0.89) | 0.028 | 0.18 (0.05 - 0.67) | 0.011 |
| TUG (s) | 1.15 (1.03 - 1.28) | 0.016 | 1.37 (1.16 - 1.62) | <0.001 |
| Handgrip strength (kgf) | 0.96 (0.93 - 0.98) | 0.001 | 0.95 (0.91 - 0.99) | 0.013 |
| 5-STS (s) | 1.01 (0.97 - 1.06) | 0.553 |  |  |
| Mini-BESTest score | 0.83 (0.76 - 0.90) | <0.001 | 0.86 (0.78 - 0.96) | 0.004 |
| Frailty | 6.02 (3.07 - 11.82) | <0.001 | 4.04 (1.67 - 9.79) | 0.002 |
| FES-I score | 1.12 (1.08 - 1.16) | <0.001 | 1.11 (1.06 - 1.17) | <0.001 |
| SF-36 questionnaire score |  |  |  |  |
| Physical functioning | 0.97 (0.96 - 0.98) | <0.001 | 0.98 (0.96 - 0.99) | 0.008 |
| Physical role | 0.98 (0.98 - 0.99) | <0.001 | 0.99 (0.98 - 0.99) | 0.016 |
| Pain | 0.99 (0.98 - 0.99) | 0.035 | 1.00 (0.99 - 1.02) | 0.706 |
| General health | 0.98 (0.96 - 0.99) | <0.001 | 0.99 (0.98 - 1.01) | 0.551 |
| Vitality | 0.98 (0.97 - 0.99) | <0.001 | 0.99 (0.97 - 1.00) | 0.080 |
| Social functioning | 1.01 (0.99 - 1.02) | 0.140 |  |  |
| Emotional role | 0.99 (0.99 - 1.01) | 0.397 |  |  |
| Mental health | 0.98 (0.97 - 0.99) | 0.001 | 0.98 (0.96 - 0.99) | 0.034 |
| Physical component summary | 0.93 (0.91 - 0.96) | <0.001 | 0.96 (0.92 - 1.00) | 0.050 |
| Mental component summary | 0.99 (0.98 - 1.01) | 0.325 |  |  |

OR = odds ratio, 95% CI = 95% confidence interval

*age, gender, educational level, body mass index body mass index categorized as obesity (greater than or equal to 30 kg/m²), history of fall, cardiovascular disease, hemoglobin < 10 g/dL, and calcium levels categorized as normocalcemia (8.8 - 10.4 mg/dL), hypocalcemia (< 8.8 mg/dL), and hypercalcemia (>10.4 mg/dL)

TUG = Timed up and go, 5-STS = 5-repetition sit-to-stand test, Mini-BESTest = Mini Balance Evaluation Systems Test, FES-I = Falls Efficacy Scale-International, SF-36 = 36-Item Short Form Health Survey
